# Skin adnexal tumor pilomatrix disease: a transformation from benign to malignant

**DOI:** 10.1093/jscr/rjaf549

**Published:** 2025-09-20

**Authors:** Salman Akthar, Mark Kranc, Matthew Mancao, Hank Hill

**Affiliations:** Bayonet Point Hospital Department of General Surgery, HCA Healthcare Florida Bayonet Point Hospital, HCA Florida Healthcare, University of South Florida Health Libraries, 14000 Fivay Rd, Hudson, FL 3466, United States; Oak Hill Hospital Department of Pathology, HCA Healthcare Oak Hill Point Hospital, HCA Florida Healthcare, University of South Florida Health Libraries, 11375 Cortez Blvd, Brooksville, FL 34613, United States; Oak Hill Hospital Department of General Surgery, HCA Healthcare Oak Hill Point Hospital, HCA Florida Healthcare, University of South Florida Health Libraries, 11375 Cortez Blvd, Brooksville, FL 34613, United States; Oak Hill Hospital Department of General Surgery, HCA Healthcare Oak Hill Point Hospital, HCA Florida Healthcare, University of South Florida Health Libraries, 11375 Cortez Blvd, Brooksville, FL 34613, United States

**Keywords:** adnexal tumors, surgical oncology, pilomatricoma, pilomatrix carcinoma

## Abstract

Skin adnexal tumors are a wide category of both benign and malignant neoplasms that exhibit morphologic differentiation into one or more than one type of adnexal structure of the skin. We present here a unique finding of a large pilomatrix carcinoma (PC) of the upper extremity in a 54 year-old-man, initially believed to be a pilomatricoma (PM). He underwent surgical excision and lymph node biopsy successfully without any complications followed by reconstructive skin grafting. Imaging and initial pathology supported a benign diagnosis, however further tissue analysis revealed a malignant PC with evidence of matrical differentiation, increased mitotic activity, and central necrosis. Our patient’s disease course may support the theory that benign PM are the origin of formation of PCs. Furthermore, this case report will discuss the histology, epidemiology, and evolving pathogenesis of these adnexal tumors.

## Introduction

Adnexal tumors, formerly known as Malherbe calcifying epitheliomas, are unique to encounter in clinical practice, with <60 reported in the literature within the last 10 years [1]. The skin is composed of three basic structures: the pilosebaceous unit, the eccrine sweat glands, and the apocrine glands, all of which derive from single cell ectoderm and the underlying mesoderm. Pilosebaceous units more specifically originate from primary epithelial germ cells and exist as basophilic cells within the basalis layer of the epidermis and protrude into the dermis as well [2]. Tumors arising from the hair follicle unit are known as pilomatricoma (PM), if benign, and pilomatrix carcinomas (PCs), if malignant. For any suspected skin adnexal tumor, accurate diagnosis is important, as there are known associations with other internal malignancies, such as trichilemmomas with Cowden disease, sebaceous tumors in Muir-Torre syndrome, and desmoid tumors in Gardner syndrome, especially if presenting with multiple synchronous lesions [3].

These tumors occur most commonly in the pediatric population, although there is thought to be a bimodal distribution, with the other spike in occurrence between ages 40 and 60 [4]. The most frequently affected bodily region is the head and neck, making up ~77% of cases, followed by the upper extremities, then lower extremities, and finally the back [5]. PMs are often freely mobile, slow-growing masses, and patients are usually asymptomatic and mistaken for epidermoid or dermoid cysts. On the other hand, PCs present as a firm, painless, violaceous nodule. It is typical for these neoplasms to have overlying ulceration as well as overlying skin discoloration.

## Case presentation

**Figure 1:**
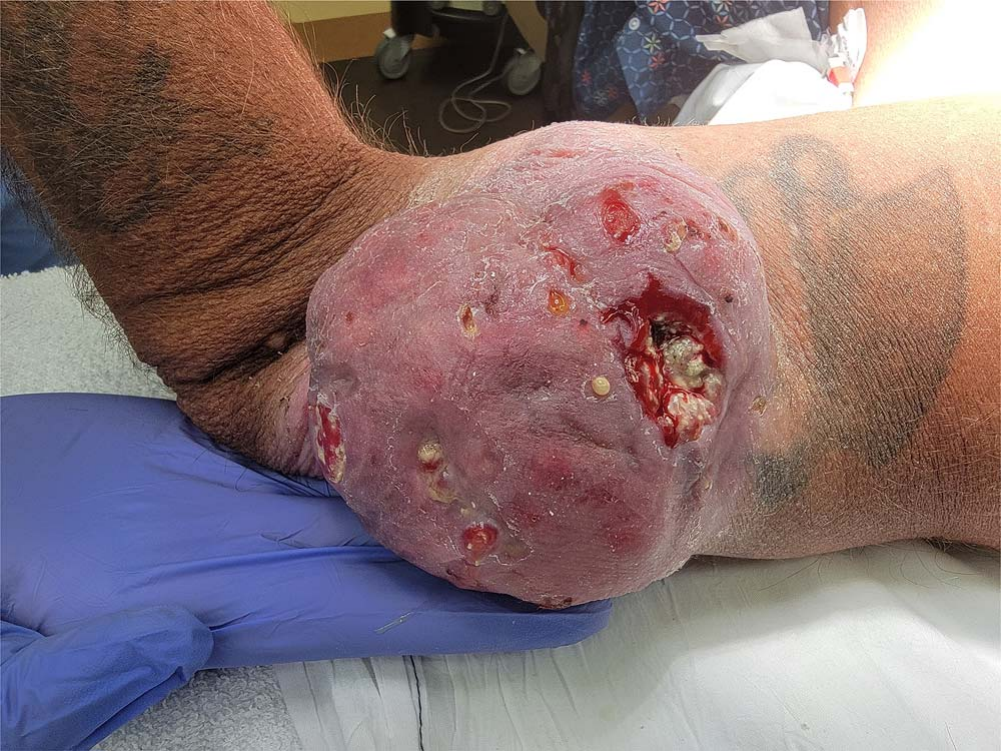
Left lateral elbow soft tissue mass, measures 11.5 × 9.5 × 4 cm.

**Figure 2:**
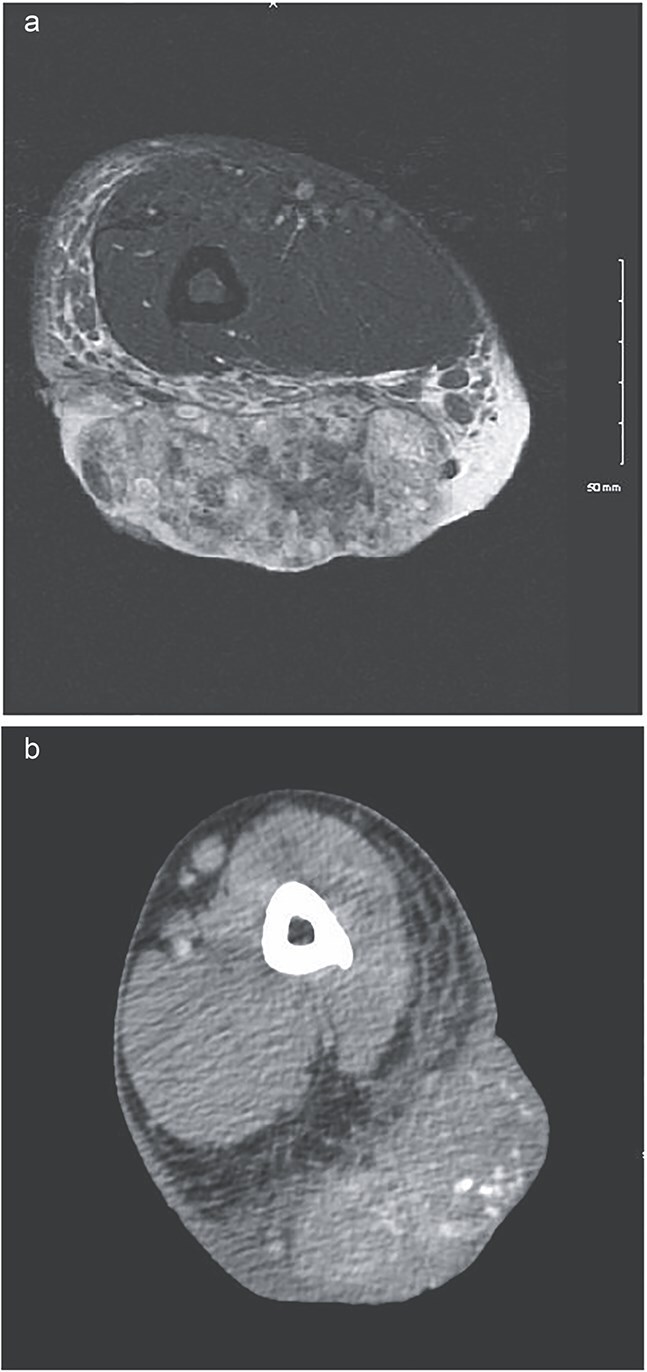
(a) MRI showing low signal on T1 and heterogeneously increased in signal on T2 sequences. Extensive surrounding subcutaneous edema with involvement of the dermal layer, as well as mild edema along the anterior fascia of the muscle. No definitive involvement of the muscle compartments noted, no involvement of neurovascular bundle. Some enlarged epitrochlear lymph nodes seen within the medial aspect of the elbow, with concern for possible local metastatic disease. (b) CT upper extremity demonstrating calcified arm lesion with vascularity supplied from branches of the proximal brachial artery. Involvement in the dermis and subdermal fat.

A 54-year-old man with a past medical history significant for deep vein thrombosis over 10 years ago, no longer on therapeutic anticoagulation, presented with a large left lateral elbow mass. It began one year prior as a small, simple lesion. Over the previous 3 months, the mass grew significantly and developed purulent drainage (Fig. 1). He had a significant tobacco use history. Family history was not significant for any familial syndromes of malignancy. Physical examination was significant for the elbow mass and ipsilateral axillary lymphadenopathy. The wound culture demonstrated mixed flora with a predominance of methicillin-resistant *Staphylococcus aureus* and *Pseudomonas aeruginosa*. Preoperative computed tomography (CT) and magnetic resonance imaging (MRI) were obtained (Fig. 2). Additional imaging, including chest CT, did not demonstrate any evidence of metastatic disease. This finding suggested no evidence of soft tissue sarcoma.

**Figure 3:**
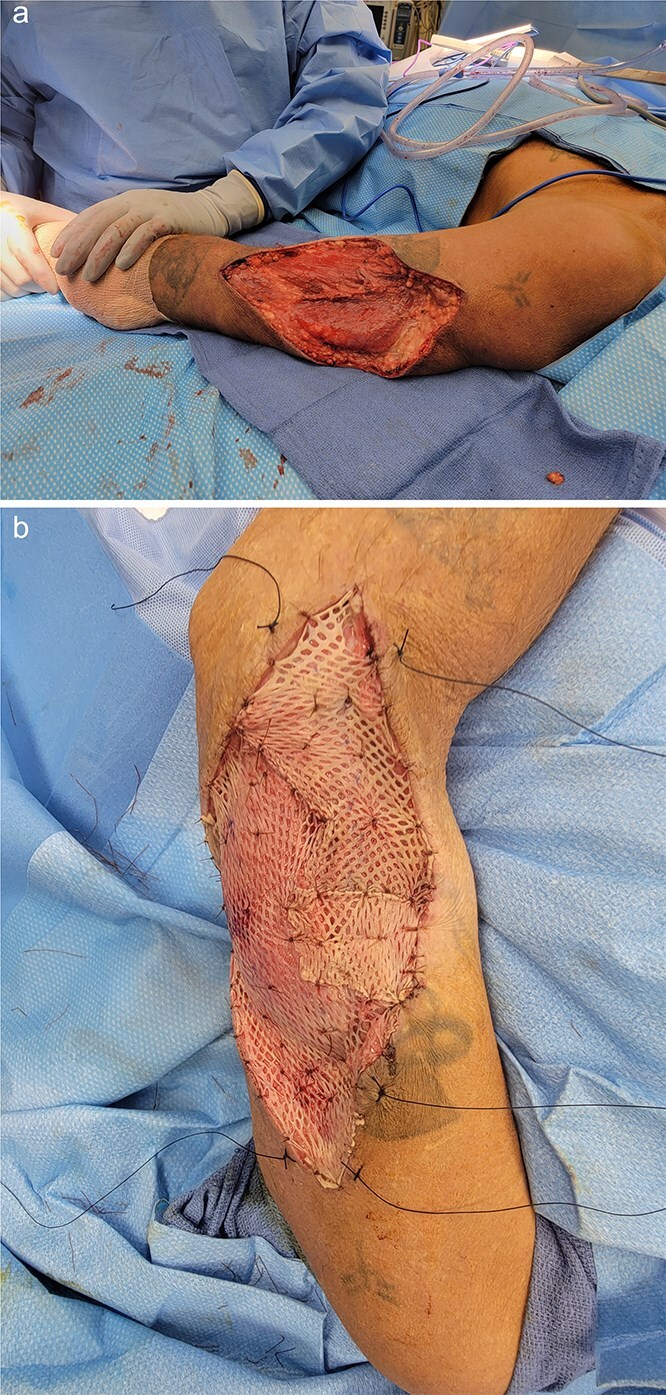
Left arm status-post surgical resection (a) followed by split-thickness skin graft application from anterior thigh donor site (b).

The patient underwent wide local excision of the soft tissue mass with sentinel lymph node biopsy (SLNB) of the axilla and elbow with Technetium Tc99m lymphoscintigraphy. A medial epitrochlear and solitary axillary node were removed. Both were sent for frozen pathology and resulted negative for malignancy. An elliptical excision was performed with 2 cm margins, and the wound was left open with black foam sponge wound vacuum-assisted closure therapy. On postoperative day 6, we performed split-thickness skin grafting from the left anterior thigh donor site (Fig. 3). He was discharged the following day.

**Figure 4:**
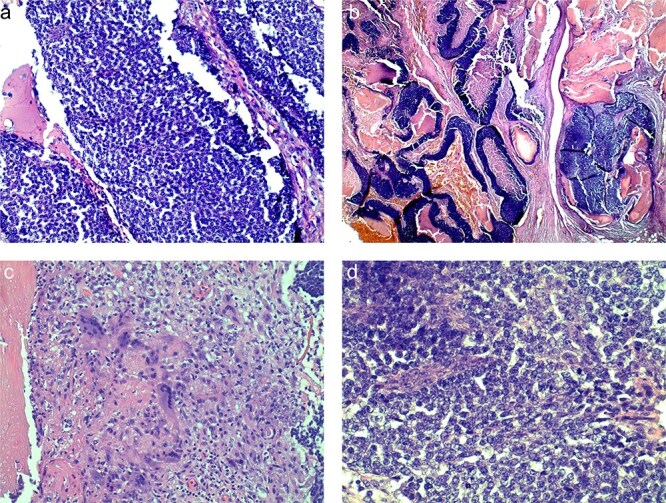
Histologic slides demonstrating basaloid cells (a), nests of basaloid and shadow cells (b), cytoplasm of ghost cells (c), shadow cells with basophilic granules (d).

The preliminary pathology report revealed SLNs were negative for any evidence of granuloma or malignancy and showed sinus histiocytosis. The soft tissue mass reported size was 11.5 × 9.5 cm and resulted benign, as a PM. His case was submitted for second opinion consultation. The final pathology report showed an infiltrative and ulcerated tumor with matrical differentiation, high proportion of matrical/germinative cells with increased mitosis, and en masse necrosis (Fig. 4). This pathology was consistent with a PC. There were clear margins and no lymphovascular invasion.

## Discussion

Differentiating between PMs and PCs can be difficult, as both types have similar epidemiology [6]. For this reason, histologic analysis is important to distinguish them. Malignancy is certain when the tumor displays poor circumscription, nuclear atypia, and atypical mitoses, areas of massive necrosis, or infiltration of the hypodermis, dermis, or cartilage [7]. Both PMs and PCs have a shared pathogenesis, thought to arise from a *CTNNB1* gene mutation. This gene, found on Chromosome 3p22.1, codes for β-catenin, a cell-signaling protein that serves in the WNT signaling pathway. A two-hit mutation in the gene leads to accumulation of β-catenin, which then translocates to the nucleus and causes upregulation of morphologic differentiation transcription factors [8]. This genetic pathway is also seen in the majority of desmoid tumors [3].

Immunohistochemistry stains, including Ki-67, CK 14, β-catenin, cyclin D1, or p53 alone or in combination, can aid in the diagnosis, although they are not always necessary. LEF1 and β-catenin staining is almost always confined to PMs and PCs, in contrast to other known skin malignancies [9]. This shared pathogenesis is important when discussing the cause of PC formation. The exact risk factors are unknown; however, he degenerating pilomatrixoma theory proposes that PMs may undergo further degeneration and transformation into PC [7]. This process is thought to be driven by a combination of genetic and epigenetic changes that result in the acquisition of malignant characteristics, often occurring after decades of a latency period [10]. Support is driven by multiple reported cases of recurrence after PMs with final pathology demonstrating a malignant lesion upon re-excision [11]. Alternatively, PCs arising de novo is difficult to support without a distinctly identified molecular pathway to differentiate between them. More studies and research are needed to understand the possibility of de novo synthesis.

Treatment for both PCs and PMs is surgical excision. Recurrence rates of PC can be as high as 50%; therefore, complete resection margins are of great importance. However, due to its rarity, the decision on appropriate margins has ranged from suggestions of 0.5 –1 cm, up to lengths of 2 cm [12]. Preoperative diagnosis of this tumor can be difficult, with an accuracy of around 30% [13]. FNA has been described as a method of aiding the clinical workup; however, accurate diagnosis is based on the histopathologic findings [14, 15]. Our recommendation, therefore, is to follow a standard diagnostic workup for all skin and soft tissue masses with core needle biopsy and/or incisional biopsy; however, excisional biopsy is also appropriate for symptomatic lesions.
